# Releasing the power of co-activation for battery ion storage

**DOI:** 10.1093/nsr/nwad202

**Published:** 2023-07-20

**Authors:** Yiying Wu

**Affiliations:** Department of Chemistry and Biochemistry, The Ohio State University, USA

**Figure 1.**
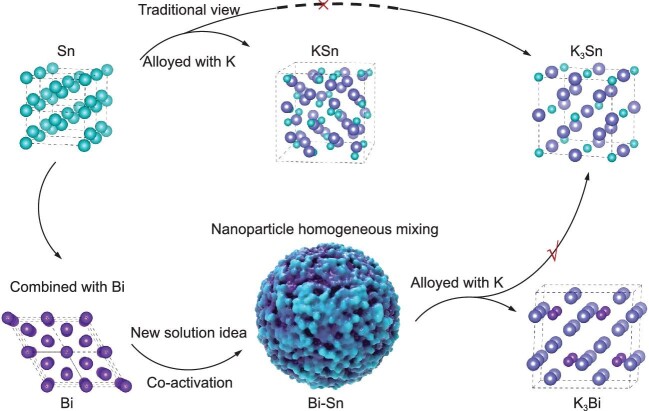
Schematic illustration of the potassiation products of Sn potassium storage, in the traditional view and the new co-activation view.

The extensive application of energy storage technology has resulted in an expanding demand for lithium resources, whose limited availability cannot support the long-term market penetration of lithium-ion batteries (LIBs). Potassium-ion batteries (PIBs) have rapidly entered the research field recently as a scalable alternative energy storage system to LIBs, relying on an abundance of potassium resources (1.5 wt% in the Earth’s crust), low-cost benefits and lower potential (−2.93 V vs. standard electrode potential) [1,2]. An essential aspect of any rising energy storage technology if it is to achieve breakthrough is that an anode is developed with both high capacity and low discharge plateau to maximize energy density and meet market demand [3]. Conventional intercalation-type anodes usually have low capacity (graphite 279 mAh g^–1^) and conversion-type anodes generally exhibit high discharge plateaus (>0.8 V) for PIBs, resulting in low energy density [4]. Alloy-type anodes, on the other hand, stand out for their high capacity, which is achieved via multiple electron transfer mechanisms and relatively low voltage plateaus. In particular, metallic tin (Sn), which is non-toxic and has low potential, exhibits remarkably high lithium storage capacity in LIBs because of a 4.4 electron transfer mechanism (Li_4.4_Sn, 993 mAh g^–1^) [5]. According to the conventional view, however, the metallic Sn anode can only transfer one electron to form KSn in PIBs (Fig. 1) and has a lower capacity (226 mAh g^–1^) than even the graphite anode (279 mAh g^–1^) [6]. Hence, stimulating the Sn’s multi-electron energy storage mechanism to obtain high capacity for PIBs has sparked much investigation among researchers. Contributing to *National Science Review*, Prof. Lu and his colleagues recently proposed a new solution: a co-activation mechanism to achieve a target anode for PIBs with excellent electrochemical performance [7].

The Bi-Sn composite anode, formed by intertwined domains of homogeneous dispersed Bi and Sn nanocrystals growing alternately along the optimal interface, was prepared by a rapid reduction method. The charge transfer at the tightly cross-linked Bi and Sn interface generates repulsive dipoles enabling an increase in the bond length of Bi-Bi and Sn-Sn bonds forming co-activation. Co-activation decreases the formation energy of potassiation products during the discharge process and consequently induces K_3_Sn formation. Traditionally, a limitation for researchers has been that Sn can only store 1 K^+^, however further analysis of the potassium storage mechanism of the Bi-Sn composite anode reveals that Bi and Sn can simultaneously store 3 K^+^ upon co-activation to form K_3_Bi and K_3_Sn, effectively enhancing the potassium storage capacity of the anode. Accordingly, the Bi-Sn anode exhibits a high potassium storage capacity of 634 mAh g^–1^ with a low discharge plateau of 0.35 V, and it lasted for 500 stable cycles at a current density of 50 mA g^–1^ with high Coulombic efficiency. In terms of full cell performance, the Prussian Blue||Bi-Sn full cell operates at 100 mA g^–1^ for 400 cycles with a capacity decay rate of only 0.023% per cycle. And the scalability of co-activation to reduce formation energy and enhance metal ion energy storage is further confirmed by Bi_0.5_-Ge composite anode sodium storage.

Unlike conventional monometallic Sn potassium storage, the co-activation mechanism between Bi and Sn proposed by Lu and his colleagues effectively addresses the previous limitations of Sn potassium storage by leveraging Bi’s activation to Sn and triggering Sn’s multi-electron transfer potassium storage potential, allowing Bi and Sn to simultaneously achieve three-electron transfer to enhance the anode’s potassium storage capacity.

Lu and colleagues’ co-activation concept introduces a groundbreaking approach to crafting high-performance anodes, exhibiting both high capacity and a low plateau. By fine-tuning the distribution ratio and reaction time, the Bi-Sn material within the composites can be optimized, resulting in nanosized components with a hyper-uniform distribution of Bi and Sn. Understanding the profound impact of co-activation at heterogeneous interfaces on ion transport kinetics is equally crucial. This insight serves as a wellspring of inspiration for the advancement of superior electrode energy storage materials, thereby propelling the practical implementation of PIB technology to new heights. The significance of this study extends far and wide, and will garner the attention of materials scientists and energy storage enthusiasts alike. With promising implications, it paves the way for the development of high-capacity PIBs and more, making it a focal point for future advancements in the field. In conclusion, Lu and colleagues’ pioneering work not only enhances our understanding of co-activation but also sets out a compelling path for the evolution of energy storage materials, fostering progress towards practical and high-capacity PIBs. As such, it holds immense potential for both scientific and technological advancements.
